# Prohemocytes are the main cells infected by dengue virus in *Aedes aegypti* and *Aedes albopictus*

**DOI:** 10.1186/s13071-022-05276-w

**Published:** 2022-04-21

**Authors:** Lie Cheng, Wei-Liang Liu, Matthew P. Su, Shu-Chen Huang, Jen-Ren Wang, Chun-Hong Chen

**Affiliations:** 1 National Mosquito-Borne Diseases Control Research Center, National Health Research Institutes, Miaoli County, Taiwan; 2 Institute of Advanced Research, Nagoya University, Nagoya, Japan; 3 Department of Biological Science, Nagoya University, Nagoya, Japan; 4 Department of Medical Laboratory Science and Biotechnology, College of Medicine, National Cheng Kung University, Tainan, Taiwan; 5 National Institute of Infectious Diseases and Vaccinology, National Health Research Institutes, Miaoli County, Taiwan

**Keywords:** Mosquito, Hemocyte, Dengue virus, *Aedes aegypti*, *Aedes albopictus*

## Abstract

**Background:**

The primary disease vectors for dengue virus (DENV) transmission between humans are the mosquitoes *Aedes aegypti* and *Aedes albopictus*, with *Ae. aegypti* population size strongly correlated with DENV outbreaks. When a mosquito is infected with DENV, the virus migrates from the midgut to the salivary glands to complete the transmission cycle. How the virus crosses the hemocoel, resulting in systemic infection, is still unclear however. During viral infection and migration, the innate immune system is activated in defense. As part of cellular-mediated immunity, hemocytes are known to defend against bacteria and *Plasmodium* infection and may also participate in defending against DENV infection. Hemocytes are categorized into three cell types: prohemocytes, granulocytes, and oenocytoids. Here, we investigated which hemocytes can be infected by DENV and compare hemocyte infection between *Ae. aegypti* and *Ae. albopictus*.

**Methods:**

Hemocytes were collected from *Ae. aegypti* and *Ae. albopictus* mosquitoes that were intrathoracically infected with DENV2-GFP. The collected hemocytes were then identified via Giemsa staining and examined microscopically for morphological differences and viral infection.

**Results:**

All three types of hemocytes were infected by DENV, though the predominantly infected cell type was prohemocytes. In *Ae. aegypti*, the highest and lowest infection rates at 7 days post infection occurred in prohemocytes and granulocytes, respectively. Prohemocytes were also the primary infection target of DENV in *Ae. albopictus*, with similar infection rates across the other two hemocyte groups. The ratios of hemocyte composition did not differ significantly between non-infected and infected mosquitoes for either species.

**Conclusions:**

In this study, we showed that prohemocytes were the major type of hemocyte infected by DENV in both *Ae. aegypti* and *Ae. albopictus*. The infection rate of prohemocytes in *Ae. albopictus* was lower than that in *Ae. aegypti*, which may explain why systemic DENV infection in *Ae. albopictus* is less efficient than in *Ae. aegypti* and why *Ae. albopictus* is less correlated to dengue fever outbreaks. Future work in understanding the mechanisms behind these phenomena may help reduce arbovirus infection prevalence.

**Graphical Abstract:**

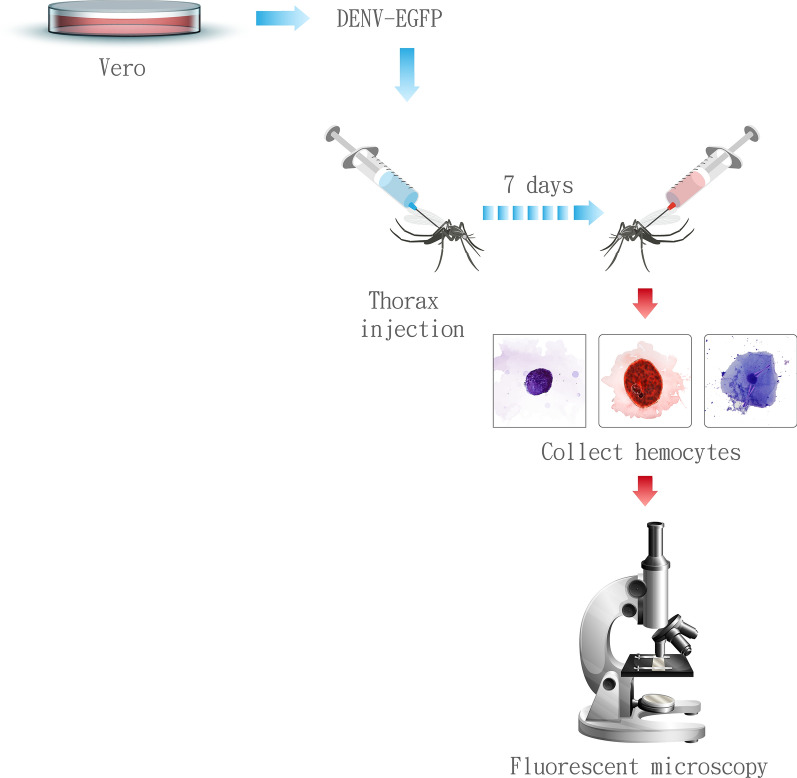

**Supplementary Information:**

The online version contains supplementary material available at 10.1186/s13071-022-05276-w.

## Background

The main vectors for dengue virus (DENV) transmission between humans are the mosquitoes *Aedes aegypti* and *Aedes albopictus*. In mosquitoes, infection with DENV starts in the midgut. After a blood meal from a host infected with DENV, the virus replicates in midgut cells and disseminates into the mosquito’s hemocoel. After circulation in the hemolymph, DENV infects other tissues in the mosquito, including the salivary glands. Once the salivary glands are infected with DENV, the infected mosquito host can spread the infection to another human with its next blood meal bite. However, how the virus travels in the hemocoel to infect other mosquito tissues remains unclear.

Historically, *Ae. aegypti* and *Ae. albopictus* have coexisted in the same geographical areas. Although *Ae. albopictus* has been responsible for dengue epidemics in Japan, Malaysia, China, and Hawaii, its vectorial capacity for DENV is lower than that of *Ae. aegypti* [1, 2]. Studies have shown that *Ae. aegypti* can be more efficiently infected with multiple DENV serotypes than *Ae. albopictus* [3], suggesting that *Ae. albopictus* are less capable of viral dissemination [3–5]. However, there is little detailed information about fundamental differences in DENV infection mechanisms between these two species.

The mosquito anti-viral defense mechanism is dependent on its activated innate immunity, which includes the Toll, IMD, JAK/STAT, and RNA interference (RNAi) pathways [6, 7]. Viruses are recognized by pattern recognition receptors (PRRs), which trigger the activation of immune pathway cascades that induce the expression of anti-microbial peptides (AMPs) or RNAi responses [8]. In addition to these well-known innate immune pathways, insects also have cellular-mediated immunity that is affected by a group of circulating macrophage-like cells referred to as hemocytes [9, 10]. Hemocytes participate in many activities in the host body, such as the promotion of tissue repair, removal of dead cells, and elimination of ingested microbes by phagocytosis, lysis, and melanization. They are also involved in the production of reactive oxygen species (ROS), reactive nitrogen species (RNS) [11], prophenoloxidase (PPO) [12], and AMPs with fat bodies, the latter of which are well-known immune cells in insects. The importance of hemocytes for bacteria and *Plasmodium* clearance is well established [13], but their role in arbovirus infection, such as DENV, is not clearly understood.

Although the mosquito host initiates immune responses to defend against DENV, the virus cannot be eliminated. Some viruses, such as human immunodeficiency virus (HIV) and human T-lymphotropic virus (HTLV-1), will infect immune cells (e.g. macrophages) to escape elimination by the immune system [14]. For example, white spot syndrome virus (WSSV) proliferates in the hemocytes of a shrimp (*Fenneropenaeus chinensis*) [15] and Sindbis virus (SINV) can infect hemocytes of *Ae. aegypti* [16]. We hypothesized that it is possible that DENV uses a similar strategy to infect the hemocytes of mosquitoes for proliferation.

There are three known types of hemocytes in mosquitoes: prohemocytes, granulocytes, and oenocytoids. Prohemocytes are round, have a high nuclear-to-cytoplasmic ratio, and are thought to be a precursor cell to granulocytes and oenocytoids [17]. They may also play a role in the infection cycle, as previous work has shown that prohemocytes are also involved in phagocytosis [18, 19]. Granulocytes are polymorphic and highly phagocytic cells. They typically have various particles or vesicles in the cytoplasm. Oenocytoids are round cells with eccentric nuclei and a homogeneous cytoplasm. They mostly produce melanin, an insoluble pigment involved in the melanization immune process [20, 21]. However, the involvement of hemocytes in arbovirus infection, including differences between types of hemocyte, still needs to be investigated.

Here, we studied the role played by hemocytes during DENV infection in both *Ae. aegypti* and *Ae. albopictus* mosquitoes. We identified differences in DENV infection in the hemocytes of these two species, which may be an important factor for the systemic infection. Further studies of this infection mechanism could provide insights into new ways to inhibit DENV spread.

## Methods

### Mosquito rearing

*Aedes aegypti* (Higgs) and *Ae. albopictus* (lab-adapted) mosquito eggs were hatched in reverse osmosis (RO) water. Larvae were transferred to larger containers and fed a mixture of yeast powder (Taiwan Sugar Corporation, Taiwan) and lyophilized powder of goose liver (#7573, NTN fishing bait LTD, Taiwan). Pupae were collected and then transferred to cages for adult emergence. Adult mosquitoes were given a constant supply of 10% sucrose water. The environment was maintained at 28 °C and 75% relative humidity under a 12-h light: 12-h dark cycle.

### DENV maintenance

DENV2-EGFP (New Guinea C strain, NGC) (Additional file 1: Fig. S1) [22] was passaged using the Vero cell line and stored in a − 80 °C freezer. Viral titers were determined via plaque assay.

### Infection of mosquitoes with DENV2

Mosquitoes were infected with DENV intrathoracically by injection of 400 PFU of DENV2 using a micro-injector (Nanoject II, Drummond Scientific Company). Injected mosquitoes were maintained in regular conditions and incubated for 7 days.

### Hemocyte collection and characterization

Hemocyte collection and characterization were performed in accordance with protocols from previous studies [23]. Each uninfected or infected mosquito was anesthetized on ice and placed on a glass slide. A pulled glass needle was filled with hemolymph diluent solution (60% Schneider’s medium, 10% fetal bovine serum (FBS), and 30% citrate buffer) and injected into the hemocoel of the mosquito in the last two abdominal segments. Injection was continued until the abdomen became filled or bloated. Injected mosquitoes were placed back on ice for 5 min. After incubation, the legs, wings, and tip of abdomen were removed from each mosquito and placed on a glass slide that was treated with 70% ethanol. Hemolymph diluent solution was injected into the thorax of the incubated mosquito using a glass needle. Hemolymph was collected by opening the abdomen. Hemolymph from 20 uninfected or infected mosquitoes was collected individually. The collected hemolymph were allowed to air-dry on the slide. The slide was fixed by treatment with methanol for 5 min and then stained with Giemsa (10 times diluted in phosphate buffer pH 7.2) for 5 min, followed by a wash with distilled water.

## Results

### Dengue virus is able to infect all three hemocyte groups in mosquitoes

To investigate whether mosquito hemocytes are infected by DENV, we first infected *Ae. aegypti* intrathoracically with DENV2–EGFP and then incubated for 7 days. Hemocytes were collected and immediately stained with Giemsa dye for visual examination. We were able to identify all three groups (prohemocytes, granulocytes, or oenocytoids) of hemocyte (Additional file 2: Fig. S2, BF). In addition, we also confirmed members of each group which were GFP positive (EGFP+) and thus presented DENV infection (Additional file 2: Fig. S2, GFP).

We found GFP-positive cells in each of the three hemocyte groups, implying that not only can DENV infect hemocytes but that it can infect all three hemocyte types. We wondered whether DENV infection differed in these distinct types and therefore analyzed the ratio of GFP positive to negative cells in each hemocyte group.

### Prohemocytes are the major hemocyte group infected with dengue virus in *Ae. aegypti*

**Fig. 1:**
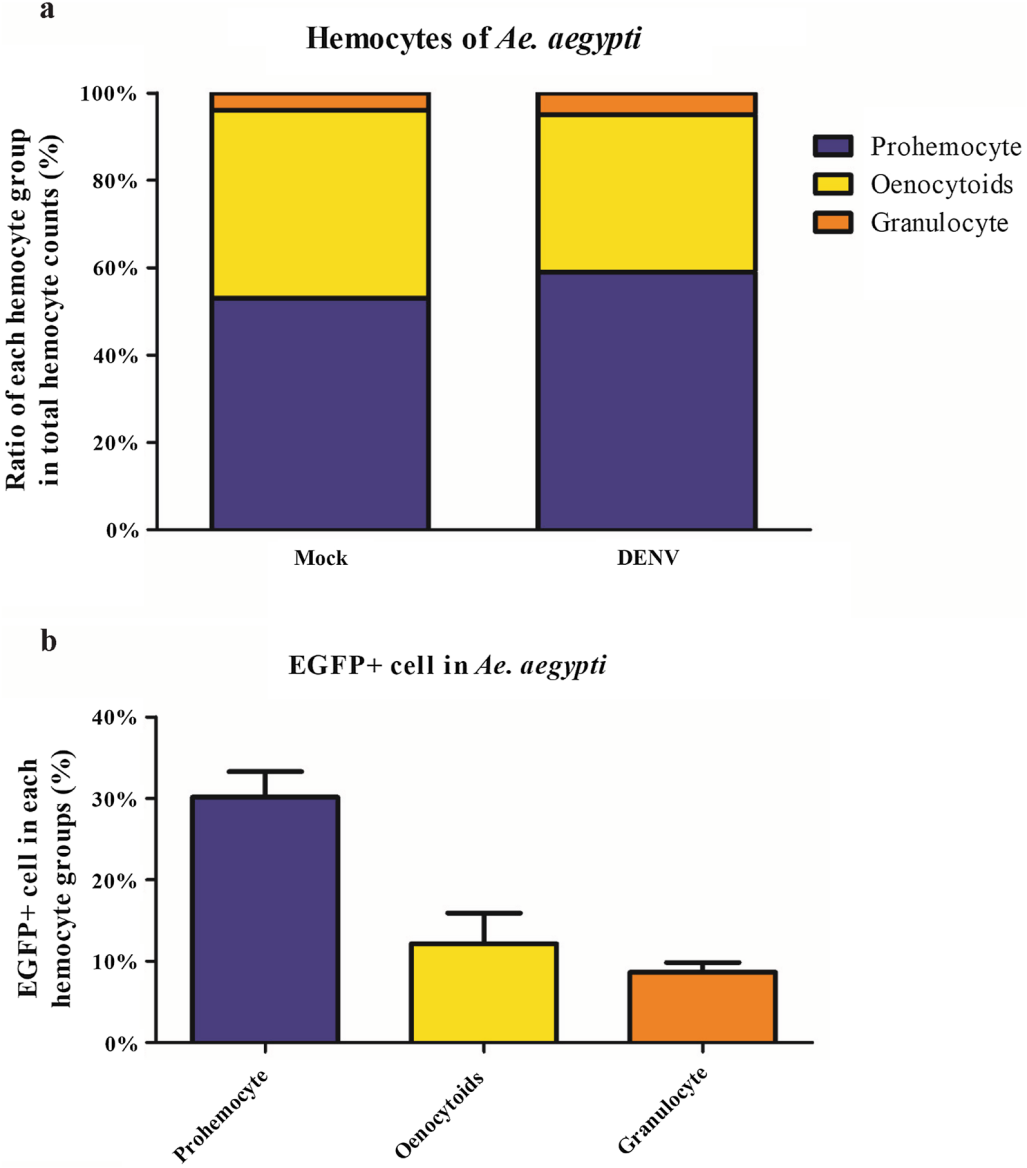
DENV infection in the hemocytes of *Ae. aegypti* adult females. Female mosquitoes were injected with DENV2-EGFP. After incubation for 7 days, hemocytes were collected, stained, and categorized by morphology into types. **a** Percentage of total collected hemocytes identified as prohemocytes, oenocytoids, or granulocytes. **b** Percentage of DENV-infected (EGFP expressing) cells in each hemocyte group

To investigate which type of hemocyte was the major target of DENV, we infected large amounts of *Ae. aegypti* intrathoracically with DENV2–EGFP. We collected more than 4000 hemocytes from at least 20 uninfected and infected mosquitoes and stained them after 7 days of incubation. We observed the cells using microscopy and determined that the ratio of hemocyte groups did not obviously change after DENV infection (Fig. 1a). We found that the prohemocytes and granulocytes represented the largest and smallest groups, respectively; prohemocytes represented above 50% of the total number of hemocytes in both infected and uninfected mosquitoes, while < 5% were granulocytes.

Next, we investigated the infection rate of DENV within each type of hemocyte. We employed the EGFP marker as a readout for DENV infection and observed that 30.1% of the prohemocytes collected from the infected mosquitoes exhibited green fluorescence (i.e. EGFP+ cell) (Fig. 1b). We also observed that 8.6% and 12.1% of granulocytes and oenocytoids, respectively, were EGFP positive (EGFP+) (Fig. 1b). From this, we concluded that prohemocytes are the major target of DENV infection in mosquito hemocytes.

### Dengue virus preferentially infects *Ae. albopictus* prohemocytes

**Fig. 2:**
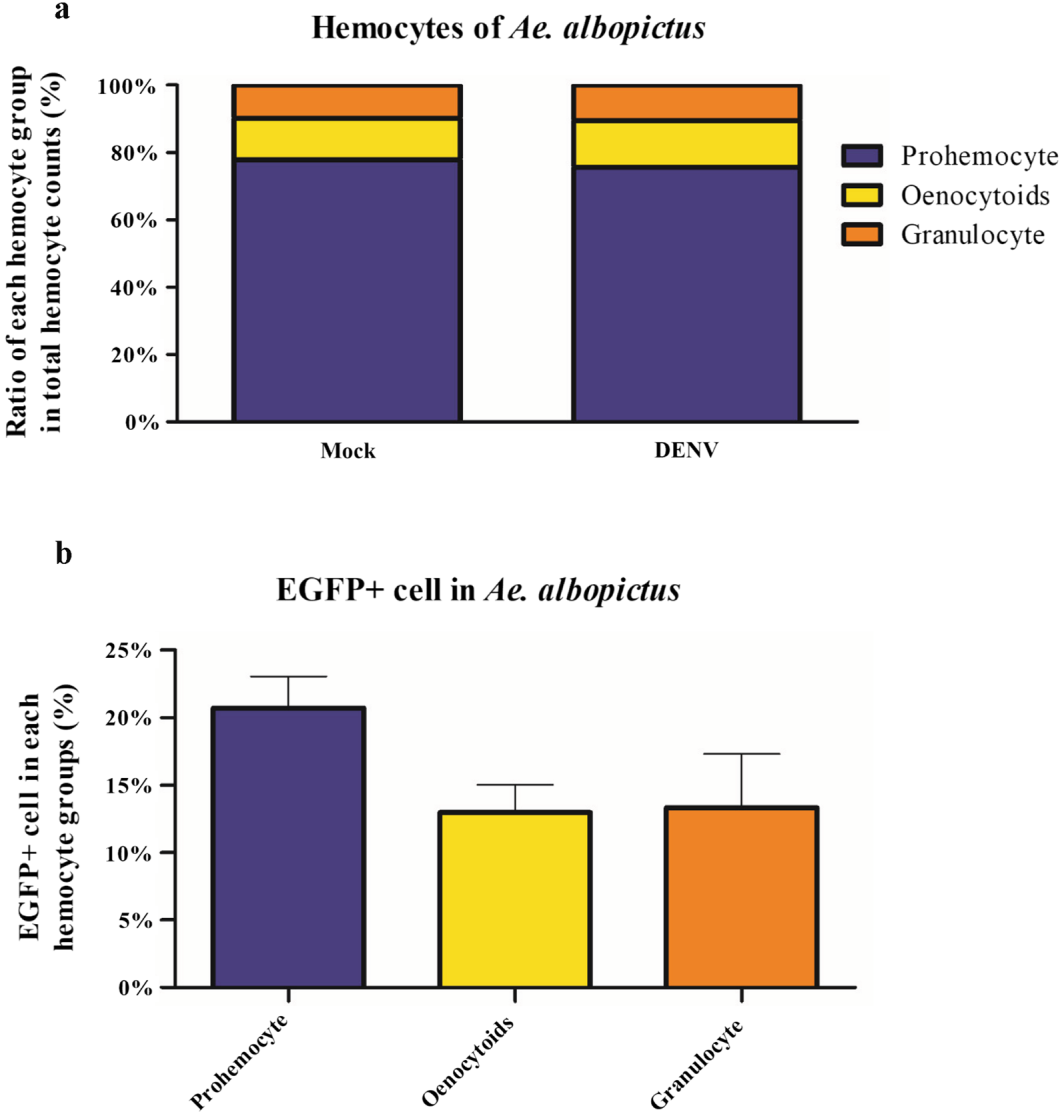
DENV infection in the hemocytes of *Ae. albopictus* adult females. Female mosquitoes were injected with DENV2-EGFP. After incubation for 7 days, hemocytes were collected, stained, and categorized by morphology into types. Infected hemocytes were confirmed with EGFP expression. **a** Percentage of total collected hemocytes identified as prohemocytes, oenocytoids, or granulocytes. **b** Percentage of DENV-infected (EGFP expressing) cells in each hemocyte group

Infection of DENV in the secondary tissue of *Ae. albopictus* is less efficient than for *Ae. aegypti* [3]. To study the infection status of *Ae. albopictus* hemocytes, we infected *Ae. albopictus* and collected and stained > 4000 hemocytes from at least 20 infected or uninfected mosquitoes. As for *Ae. aegypti*, we found that the largest group of hemocytes in *Ae. albopictus* was prohemocytes, at > 70% of the total hemocyte population in infected and uninfected mosquitoes (Fig. 2a). Granulocytes were the smallest group, at < 12% of the total population.

We also analyzed the DENV infection rate in individual hemocyte groups. In prohemocytes of infected *Ae. albopictus*, we found that 20.7% of cells were infected with DENV (Fig. 2b). In granulocytes and oenocytoids, 13.0% and 13.3% of cells, respectively, were infected with DENV (Fig. 2b). Taken together, these results suggest that DENV does infect hemocytes in *Aedes* mosquitoes and predominantly targets prohemocytes. In addition, DENV infection rates of hemocytes are lower in *Ae. albopictus* than in *Ae. aegypti*.

## Discussion

*Ae. albopictus* and *Ae. aegypti* are both major vectors of DENV, though their relative vector competences are drastically different. Though the two species share similar physiologies, small differences may be sufficient to explain these distinct vector competences, potentially via differential infection of hemocytes. In this study, we address the question of how DENV travels in mosquito bodies to infect other tissues by demonstrating that DENV infected and replicated in the hemocytes of both intrathoracically infected mosquito vectors. Our results also showed a stronger infection presence in *Ae. aegypti*, which may indicate it is the more potent vector.

Our work here demonstrated that hemocytes are indeed infected by DENV in mosquitoes, though not equally across all cell types. Among the three types of hemocytes, prohemocytes had the highest infection rate, suggesting that a different function of prohemocytes makes them more likely to encounter the virus or become infected. The other types of hemocytes, granulocytes and oenocytoids, were infected by DENV as well. A recent study using single-cell RNA sequencing (scRNA-seq) suggested these two hemocyte types may be divided into different sub-groups with distinct functions [24]. The reasons for the preferential infection of prohemocytes, and whether sub-groups of the other hemocytes are differentially targeted by DENV, require further investigation.

We also identified DENV replication in cells other than prohemocytes with DENV2–EGFP, which might indicate that those other cells also supported DENV replication but at lower levels. Alternatively, since prohemocytes are thought to be the precursor cells of granulocytes and oenocytoids [17], we hypothesize that the infection of these cells may come before their differentiation. For instance, when infected prohemocytes differentiate into granulocytes or oenocytoids, the differentiated daughter cells may still be infected with DENV. This hypothesis would also benefit from a deeper investigation into the mechanism of infection of prohemocytes versus other cell types.

To further probe the reasons for prohemocytes having the highest ratio of infection with DENV, we speculated on possible mechanisms of infection that could be investigated in future studies. Prohemocytes, as well as granulocytes, are highly phagocytic cells, and phagocytosis can be rapidly initiated when the cells are exposed to pathogens. Previous studies in mammalian cells showed that cell surface phagocytosis receptors, such as dendritic cell-specific intercellular adhesion molecule-3-grabbing non-integrin (DC-SIGN) and mannose receptor (MR) [25–27], are involved in DENV entry. We suspect DENV could infect mosquito prohemocytes through a similar mechanism because of the role of prohemocytes in phagocytosis. The possible role of phagocytosis in DENV infection is also strengthened by considering the overall function of phagocytosis as well as differences in this function between midgut and systemic infections [28]. For instance, orally infected mosquitoes have an increased number of hemocytes recruited to the midgut. When phagocytosis is blocked in hemocytes, the midgut infection is less severe, but the systemic infection is intensified. The type of hemocyte involved in these two kinds of infections has not yet been specified, so we cannot be sure which type of hemocyte was involved in these differences. We speculate that prohemocytes are the major hemocyte type involved in defending the midgut tissue against DENV infection and granulocytes are the major type involved in systemic infection, as granulocytes are considered to be a typical phagocytic hemocyte for bacterial infection. If prohemocytes localized to the midgut to combat DENV infection via phagocytosis and were then infected, the prolonged localization of prohemocytes in the midgut tissue would aggravate midgut infection.

Our study did contain an anomaly compared to previous studies—we obtained 150–250 hemocytes per adult female mosquito, which was lower than that of previous studies (around 500–4000 hemocytes per adult mosquito [17]). One possible reason is the natural decrease in hemocytes with adult age in mosquitoes (days). We allowed 4–5 days for the mosquitoes to achieve maturation and mating before intrathoracically infecting them with DENV. After we infected the mosquitoes intrathoracically, we incubated them for an additional 7 days to achieve systemic infection. Adult mosquitoes that are 11–12 days old were shown to have about half the number of hemocytes than 1–3 days old adult mosquitoes [17]. Therefore, we assume that the number of granulocytes, oenocytoids, and prohemocytes will differ between mosquitoes that are 1 and 12 days old.

Finally, our study showed that the ratio of infected prohemocytes in *Ae. albopictus* was lower than that in *Ae. aegypti*, whereas the infection rates for granulocytes and oenocytoids were similar. Therefore, *Ae. aegypti* could have greater infection rates in secondary tissues across the hemocoel and midgut than *Ae. albopictus*. The mechanisms behind the lower virulence of DENV and lack of correlation to outbreaks in *Ae. albopictus* are unclear and can be contradictory between studies. Our work here matches previous work showing that infection by multiple serotypes of DENV in *Ae. albopictus* is less efficient than in *Ae. aegypti* [3]. Furthermore, studies have reported lower dissemination of DENV-2 to the secondary tissues (e.g. Malpighian tubules, ovaries, and salivary glands) of *Ae. albopictus* than *Ae. aegypti* [3, 5]. Consequently, this may decrease the risk of DENV transmission via *Ae. albopictus* to other human hosts. However, other studies indicate that this may not be affected by the activation of immune pathways. Toll and JAK/STAT immune pathways, important pathways in defending against virus infection, were equally activated in response to DENV infection in both species [29]. Another possible reason for lower DENV virulence in *Ae. albopictus* may be its role as a natural host of *Wolbachia*, a bacteria that naturally occurs in some insects. *Wolbachia* has artificially been introduced to *Ae. aegypti* to combat DENV transmission for several years, with this having been shown to reduce infection and dissemination in *Aedes* species [30].

## Conclusions

In this study, we collected and examined the hemocytes of *Ae. aegypti* and *Ae. albopictus* intrathoracically infected with DENV. Our finding that prohemocytes were the primary targets of DENV infection, with over 20% infected, sheds new light on the mosquito infection process, which is key for discovering ways to reduce later transmission to humans. The greater proportion of prohemocytes infected in *Ae. aegypti* over *Ae. albopictus* may be a factor for lower DENV transmission between human hosts that are bitten by *Ae. albopictus.* These data may explain the lower correlation between *Ae. albopictus* and dengue fever outbreaks in endemic areas. As the major component of cellular-mediated immunity, hemocytes may play a crucial role in systemic DENV infection. Further understanding of the detailed mechanisms of viral infection in hemocytes may be helpful in generating novel methods of reducing DENV transmission.

## Supplementary Information

**Additional file 1: Figure S1.** Schematic representation of DENV2-EGFP. The EGFP gene was inserted into the DENV capsid (C) for production during viral replication.

**Additional file 2: Figure S2.** Hemocytes of *Ae. aegypti* can be infected with DENV. Adult female mosquitoes were injected with DENV2-EGFP, which is used as a fluorescent indicator for DENV infection. Hemocytes were collected from uninfected (left panel) and infected (right panel) mosquitoes, and their morphologies observed with 100× phase contrast (BF) and green channel (GFP) fluorescence microscopy, shown on the left and right of each pair of images, respectively. Hemocytes can be divided into three groups based on their observed morphology under fluorescent microscopy and Giemsa staining: prohemocytes, oenocytoids, and granulocytes.

## Data Availability

The datasets supporting the findings of this article are included within the article and its Additional files.

## Abbreviations

DENV: Dengue virus
*Ae. aegypti*: *Aedes aegypti*
*Ae. albopictus*: *Aedes albopictus*
IMD: Immune deficiency
JAK/STAT: Janus kinase/signal transducer and activator of transcription
RNAi: RNA interference
PRRs: Pattern recognition receptors
AMPs: Anti-microbial peptides
ROS: Reactive oxygen species
RNS: reactive nitrogen species
PPO: Prophenoloxidase
HIV: Human immunodeficiency virus
HTLV-1: Human T-lymphotropic virus
WSSV: White spot syndrome virus
SINV: Sindbis virus
RO: Reverse osmosis
NGC: New Guinea C strain
PFU: Plaque-forming unit
FBS: Fetal bovine serum
EGFP: Enhanced green fluorescent protein
scRNA-seq: Single-cell RNA sequencing
DC-SIGN: Dendritic cell-specific intercellular adhesion molecule-3-grabbing non-integrin
MR: Mannose receptor
