# Supplementary figures and images for: Prohemocytes are the main cells infected by dengue virus in Aedes aegypti and Aedes albopictus
Source: Parasit Vectors. 2022 Apr 21;15:137. doi: 10.1186/s13071-022-05276-w (PMC9027048; doi:10.1186/s13071-022-05276-w)

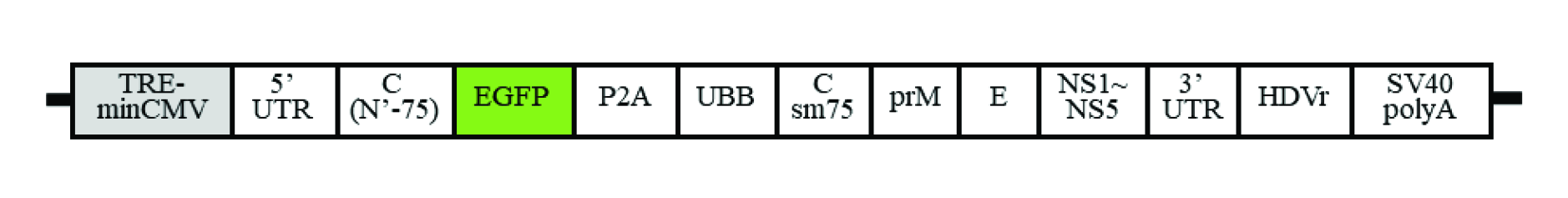

Supplement: Supplementary file 1 — Additional file 1: Figure S1. Schematic representation of DENV2-EGFP. The EGFP gene was inserted into the DENV capsid (C) for production during viral replication. [file 13071_2022_5276_MOESM1_ESM.tiff]

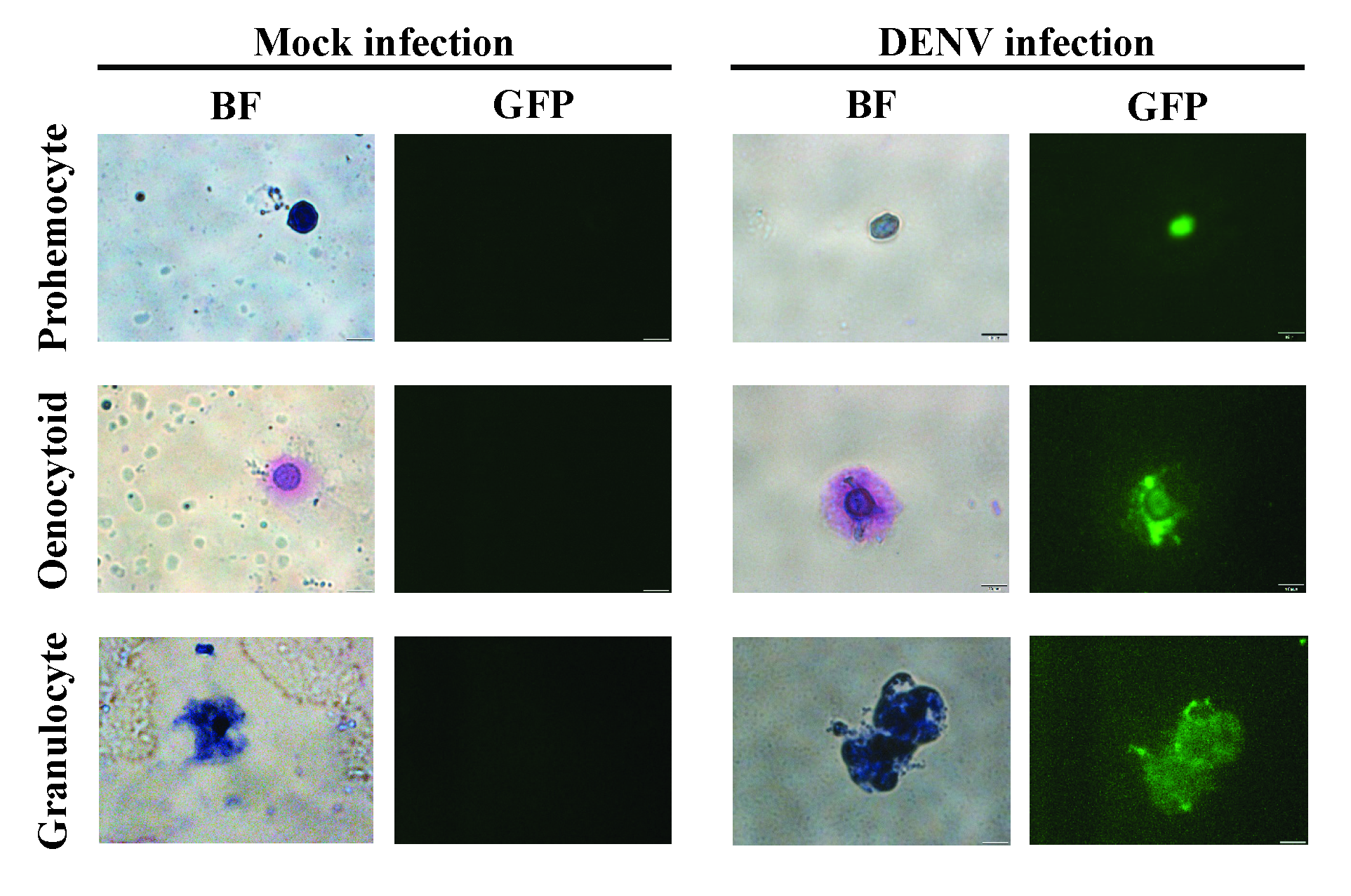

Supplement: Supplementary file 2 — Additional file 2: Figure S2. Hemocytes of Ae. aegypti can be infected with DENV. Adult female mosquitoes were injected with DENV2-EGFP, which is used as a fluorescent indicator for DENV infection. Hemocytes were collected from uninfected (left panel) and infected (right panel) mosquitoes, and their morphologies observed with 100× phase contrast (BF) and green channel (GFP) fluorescence microscopy, shown on the left and right of each pair of images, respectively. Hemocytes can be divided into three groups based on their observed morphology under fluorescent microscopy and Giemsa staining: prohemocytes, oenocytoids, and granulocytes. [file 13071_2022_5276_MOESM2_ESM.tiff]
